# Expression and Physiology of Voltage-Gated Sodium Channels in Developing Human Inner Ear

**DOI:** 10.3389/fnins.2021.733291

**Published:** 2021-10-25

**Authors:** Rikki K. Quinn, Hannah R. Drury, Ethan T. Cresswell, Melissa A. Tadros, Bryony A. Nayagam, Robert J. Callister, Alan M. Brichta, Rebecca Lim

**Affiliations:** ^1^School of Biomedical Sciences and Pharmacy, The University of Newcastle, Callaghan, NSW, Australia; ^2^Hunter Medical Research Institute, The University of Newcastle, New Lambton Heights, NSW, Australia; ^3^Department of Audiology and Speech Pathology, The University of Melbourne, Parkville, VIC, Australia

**Keywords:** vestibular, cochlea, human, development, PCR, sodium channel, electrophysiology, inner ear

## Abstract

Sodium channel expression in inner ear afferents is essential for the transmission of vestibular and auditory information to the central nervous system. During development, however, there is also a transient expression of Na^+^ channels in vestibular and auditory hair cells. Using qPCR analysis, we describe the expression of four Na^+^ channel genes, SCN5A (Nav1.5), SCN8A (Nav1.6), SCN9A (Nav1.7), and SCN10A (Nav1.8) in the human fetal cristae ampullares, utricle, and base, middle, and apex of the cochlea. Our data show distinct patterns of Na^+^ channel gene expression with age and between these inner ear organs. In the utricle, there was a general trend toward fold-change increases in expression of SCN8A, SCN9A, and SCN10A with age, while the crista exhibited fold-change increases in SCN5A and SCN8A and fold-change decreases in SCN9A and SCN10A. Fold-change differences of each gene in the cochlea were more complex and likely related to distinct patterns of expression based on tonotopy. Generally, the relative expression of SCN genes in the cochlea was greater than that in utricle and cristae ampullares. We also recorded Na^+^ currents from developing human vestibular hair cells aged 10–11 weeks gestation (WG), 12–13 WG, and 14+ WG and found there is a decrease in the number of vestibular hair cells that exhibit Na^+^ currents with increasing gestational age. Na^+^ current properties and responses to the application of tetrodotoxin (TTX; 1 μM) in human fetal vestibular hair cells are consistent with those recorded in other species during embryonic and postnatal development. Both TTX-sensitive and TTX-resistant currents are present in human fetal vestibular hair cells. These results provide a timeline of sodium channel gene expression in inner ear neuroepithelium and the physiological characterization of Na^+^ currents in human fetal vestibular neuroepithelium. Understanding the normal developmental timeline of ion channel gene expression and when cells express functional ion channels is essential information for regenerative technologies.

## Introduction

Voltage-gated sodium channels are essential for the propagation of action potentials in neurons. There are nine different α-subunits in Na^+^ voltage-gated ion channels that are expressed in the central and peripheral nervous systems that are critical for neuronal excitability and function (Wang et al., 2017). In sensory afferent fibers there are different patterns of Na^+^ channel subunit expression during development, that likely influences the sensitivity and firing patterns of these neurons (Thun et al., 2009; Tadros et al., 2015; Kim and Rutherford et al., 2016; Meredith and Rennie, 2020). In the central nervous system, there is a developmentally regulated expression of sodium channel genes specifically targeting excitatory or inhibitory neurons which is likely to establish distinct firing rates (Du et al., 2020). In addition to the essential role in action potential discharge, Na^+^ channel expression during development has a role in synaptogenesis (Chabbert et al., 2003; Brugeaud et al., 2007; Zu et al., 2021) and may be important for cell differentiation and afferentation (Oliver et al., 1997; Wooltorton et al., 2007).

There is a diversity in the expression of Na^+^ channel genes including Nav 1.1–Nav 1.9 in the inner ear at different stages of development (Mechaly et al., 2005; Wooltorton et al., 2007; Fryatt et al., 2009; Frucht et al., 2011; Yoshimura et al., 2014; Liu et al., 2016), that is thought to be important for establishing appropriate neural connections. The β-subunits of voltage-gated Na^+^ channels, encoded by SCNB genes have also been tracked in the developing the inner ear (Wooltorton et al., 2007; Liu et al., 2016). Complementing these molecular results are anatomical investigations that have documented the expression and location of various Na^+^ channel α-subunits within hair cells, afferent terminals, and ganglia of the auditory and vestibular systems (Wooltorton et al., 2007; Lysakowski et al., 2011; Eckrich et al., 2012; Kim and Rutherford et al., 2016; Liu et al., 2016; Zhou et al., 2020). Functional studies have characterized the presence of different types of Na^+^ currents (attributable to α- and β-subunits) in both developing and mature vestibular and auditory hair cells and ganglion neurons of rat, mouse, and gerbil (Santos-Sacchi, 1993; Rüsch and Eatock, 1996; Chabbert et al., 1997; Marcotti et al., 2003; Geleoc et al., 2004; Wooltorton et al., 2007; Li et al., 2010; Liu et al., 2016; Browne et al., 2017). In vestibular type I and type II hair cells, there is evidence for the presence of both tetrodotoxin (TTX)-insensitive and TTX-sensitive Na^+^ channels, respectively, and that Na^+^ channel expression in utricle is developmentally regulated (Wooltorton et al., 2007). By postnatal day (PND) 21, there is a loss of TTX-sensitive Na^+^ channels in type II hair cells and a significant down-regulation of TTX-insensitive Na^+^ channels in type I hair cells (Wooltorton et al., 2007). This transient Na^+^ channel expression is reduced after synaptogenesis and the establishment of afferent contacts (Wooltorton et al., 2007). In the cochlea, the expression of Na^+^ channels during development in outer hair cells (OHCs) may be linked to establishing and refining afferent and efferent connections (Oliver et al., 1997). In inner hair cells (IHCs), during embryonic and early postnatal development (embryonic E16.5 – PND 12), there is a transient expression of Na^+^ channels that are responsible for modulating spike frequency of Ca^2+^-evoked action potentials (Marcotti et al., 2003; Eckrich et al., 2012). With the onset of hearing (after PND 12 in mice), there is a down-regulation of these Na^+^ channels in IHCs.

The expression of voltage-gated Na^+^ channels in auditory and vestibular ganglion neurons is essential for action potential generation and propagation of auditory and vestibular information from the periphery to the central nervous system. Vestibular ganglion neurons, like afferent fibers have a diversity of spike frequencies and timing, i.e., regular versus irregular discharge, which is likely due to the expression of different Na^+^ channels including persistent and resurgent Na^+^ channels (Liu et al., 2016; Meredith and Rennie, 2018, 2020). Similarly, the high frequency of auditory afferent discharge and requirement for precise timing and localization of auditory inputs is dependent on the expression of a combination of TTX-sensitive, TTX-insensitive, persistent, and resurgent Na^+^ channels in spiral ganglion neurons (Santos-Sacchi, 1993; Browne et al., 2017).

In summary, our current understanding of Na^+^ channel expression in the auditory and vestibular systems has arisen almost exclusively from developing and mature animal models. Some work on human tissue has described ion channel expression, however, this has been restricted to immunolabelling in diseased adult tissue (Hotchkiss et al., 2005). No studies have characterized the expression and few have described the function of Na^+^ channel physiology in developing human hair cells (Lim et al., 2014). To address this deficit, we examined mRNA expression of four Na^+^ channel types that are known to be functionally important in cochlea and vestibular end organs during embryonic and postnatal development in other species. We also characterize Na^+^ channel physiology in developing vestibular hair cells using patch clamp electrophysiology. Our experimental approach uses human fetal inner ear neuroepithelia aged between 10 and 17 WG, a period of human inner ear development when rapid changes are occurring (see review Lim and Brichta, 2016).

## Materials and Methods

### Tissue Collection

Tissue samples were obtained from elective terminations (10–17 WG). All procedures were approved by The University of Newcastle Human Ethics Committee and complied with Australia’s National Health and Medical Research Council, National Statement on Ethical Conduct in Human Research regulations. Written consent was obtained from all donors and no identifying information was supplied to researchers. No samples were collected with known medical or genetic anomalies. Gestational age was determined by three criteria: (1) date of last menstrual period, (2) ultrasound measurement of crown – rump length, and (3) foot length (Hern, 1984). Products of conception (POC) were collected in a cold glycerol-based artificial cerebrospinal fluid (ACSF) containing (in mM); 250 glycerol, 26 NaHCO_3_, 11 glucose, 2.5 KCl, 1.2 NaH_2_PO_4_, 1.2 MgCl_2_, and 2.5 CaCl_2_ and then transported to a PC2 laboratory at The University of Newcastle. All samples arrived at our laboratory within 60 min of the termination procedure. All experiments were also approved by the University of Newcastle Institutional Biosafety Committee.

### Tissue Preparation

Inner ears were obtained from POC and transferred to a dissecting well filled with fresh glycerol-based ACSF that was bubbled with 5% CO_2_/95% O_2_ as previously described (Lim et al., 2014). At the developmental stages we examined (10–17 WG), the precursor of the bony labyrinth is cartilaginous and not calcified. Consequently, it was peeled away to expose the underlying membranous labyrinth. The neuroepithelium of the vestibular organs (cristae ampullares and utricle) and cochlea were micro dissected and the membranes overlying the neuroepithelium were removed. The vestibular organs were used for either molecular biology analysis or electrophysiological experiments. For molecular biology experiments, the vestibular organs (cristae ampullares, utricle) and the cochlea were transferred to a vial of RNAlater (Merck, Australia), stored at 4°C overnight and then at −20°C for processing at a later date. Hair cell layers were not further dissected in either cochlea or vestibular organs. Thus, our qPCR results measure gene expression from epithelium containing hair cells, afferent and efferent terminals, and stromal cells. For electrophysiology experiments, whole vestibular organs were transferred to a recording chamber (Lim et al., 2014) containing oxygenated Liebovitz’s L15 cell culture medium containing (in mM); 1.26 CaCl_2_, 0.98 MgCl_2_, 0.81 MgSO_4_, 5.33 KCl, 44 KH_2_PO_4_, 137.93 NaCl, 1.34 NaH_2_PO_4_ (Life Technologies, Australia; pH 7.45, 305 mOsM), and perfused at an exchange rate of 3 ml/min.

### RNA Extraction

For each sensory region (cristae ampullares, utricle, and cochlea) from an individual POC sample aged 10–17 WG, RNA was extracted using QIAGEN miRNeasy Kit (QIAGEN, Australia) according to manufacturer’s instructions. Briefly, sensory regions were homogenized in 700 μl QIAzol lysis reagent and incubated at room temperature for 5 min. Samples were loaded into QIAGEN miRNeasy spin columns and total and small RNA were isolated. RNA quality and quantity were determined using Nanospectrophotometry.

### DNase I Treatment

Total RNA (60–200 ng) was treated with 1 μl DNase I (ThermoFisher Scientific, Australia), 1 μl DNase I buffer, and molecular biology grade water to total volume = 10 μl and incubated at room temperature for 15 min. Next, 1 μl of 25 mM EDTA was added and samples incubated for 10 min at 65°C.

### Reverse Transcription

Reverse transcription was performed using SuperScript III (ThermoFisher Scientific, Australia), according to the manufacturer’s instructions. Briefly, 30–100 ng of total RNA, 1 μl oligo(dT)18 primers, 1 μl of random hexamer, 1 μl of 10 mM dNTP, and molecular biology grade water to total volume = 13 μl, were mixed and heated for 5 min at 65°C. After incubation, 4 μl 5× first-strand buffer, 1 μl of 0.1 M DTT, 1 μl RiboSafe RNase Inhibitor (40 U/μl) and 1 μl SuperScript III RT (200 U/μl) were added and samples incubated for 60 min at 50°C, followed by 15 min at 70°C.

### qPCR

All qPCR primers were designed using NCBI Primer-BLAST (see Table 1). Primer efficiencies, specificity, and optimal cDNA quantities were determined using serial dilutions of neuronal cDNA from human POCs. Reactions contained 6.25 μl 2× SensiFAST SYBR^®^ Lo-ROX (Bioline, Australia), 1–4 ng cDNA, 200 nm each of forward and reverse primers and molecular biology grade water to total volume = 12.5 μl. Reactions were performed in triplicate on an ABI 7500 Real-Time PCR System (Applied Biosystems, United States) and analyzed using the Applied Biosystems 7500 Sequence Detection software (version 1.4). Delta Ct (ΔCt, threshold cycle) was determined for each gene relative to the housekeeping gene β-Actin (ACTB). Expression of a second housekeeping gene (GAPDH) was also done. Our analysis found there was a potential age-related change in GAPDH in inner ear samples. Consequently, GAPDH was not used as a reference gene, rather, β-actin was used for sample normalization. To confirm the use of β-actin as a reliable and valid housekeeping gene with stable expression, the entire data set was screened using RefFinder (Xie et al., 2012). The ΔΔCt method was employed to compare gene expression across the three different age groups (10–11 WG, 12–13 WG, and 14+ WG).

**TABLE 1:** Primer sequences for SCN5A, SCN8A, SCN9A, and SCN10A.

**Gene**	**Forward sequence**	**Reverse Sequence**	**Primer efficiency**
SCN5A	GAGTACACCTTCAC CGCCAT	TCACACTAAAGTCCA GCCAGTT	93.7%
SCN8A	ATTTGAAGGGATGAG GGTGGTG	AAGCAGTAGTGGTA CTTTCCC	105.8%
SCN9A	ACCCCCAATCAGTC ACCACT	GACTTGTTCTGCTG CTTCGC	83.0%
SCN10A	TCGCTAATCCGACT GTGTGG	TCCTGCTGTCCTTTG GGGAT	106.1%

### Data Presentation and Statistical Analysis

Plots of ΔΔCt for each gene were generated by normalizing data in each tissue sample to 10–11 WG values and represented as ***fold change*** to compare developmental changes. Comparisons of gene expression between tissue samples (i.e., utricle versus base of cochlea) for a single age group are described as ***relative expression*** to the housekeeping gene, β-actin.

In figures that show fold change, each data point represents the mean fold change ± SEM (fold change ratio) for the gestational ages shown.

As our data were not normally distributed, differences were described by relative expression of the median and were analyzed by the Mann–Whitney *U* test. Effect size following Mann–Whitney *U* test was calculated by the following formula:


$$\eta^{2}={\text{Z}}^{2}/\text{N}$$

Effect size was calculated to compare relative gene expression across ages and tissue regions. An advantage of calculating effect size is the independence from sample size (Schafer and Schwarz, 2019). This increases the likelihood of detecting biologically relevant results within a small sample set. Effect sizes for η^2^ are η^2^ > 0.01 = small effect size, η^2^ > 0.06 = medium effect size, and η^2^ > 0.14 = large effect size (Cohen, 1988). Effect sizes of median gene expression were calculated between the three age groups for each tissue and between tissues at each age group.

### Electrophysiological Recordings

Whole cell patch clamp recordings of voltage-gated Na^+^ channel activity were made from tissue samples aged between 10 and 16 WG. Hair cell recordings began within 90–120 min from the time of the termination procedure and tissue was viable for up to 5 h post-procedure. Whole vestibular organs (individual crista, utricle, or vestibular triad) were transferred to the recording chamber. Borosilicate glass pipettes (3–5 MΩ) were used and filled with KCl/gluconate based internal solution. The KCl/gluconate based internal solution contained (in mM); 42 KCl, 98 K-gluconate, 4 HEPES, 0.5 EGTA, 1 MgCl_2_, 5 Na-ATP, and pH 7.3 (Poppi et al., 2018). All recordings were made at room temperature (22–25°C). Hair cells were recorded from the explant and identified using either a Zeiss Axioscope 2 FS or Olympus BX51WI microscope with infra-red differential interference contrast optics. In some experiments TTX (Alomone, Israel; 1 μM) and cadmium (Sigma Aldrich, Australia; 300 μM) were added to the perfusate. Recordings were collected using either an Axopatch 1D or MultiClamp 200B amplifier, both using Axograph X software. Data were sampled at 20 kHz and filtered at 2–10 kHz. Series resistance (*R*_*s*_) was monitored throughout the recording sessions and data were rejected if *R*_*s*_ changed by more than 20%. The mean *R*_*s*_ was 14.3 ± 1.4 MΩ. Data were recorded from hair cells of both the cristae and utricle. Data are presented as mean ± SEM. Data were analyzed by Mann–Whitney *U* statistical test since data were not normally distributed.

## Results

**FIGURE 1:**
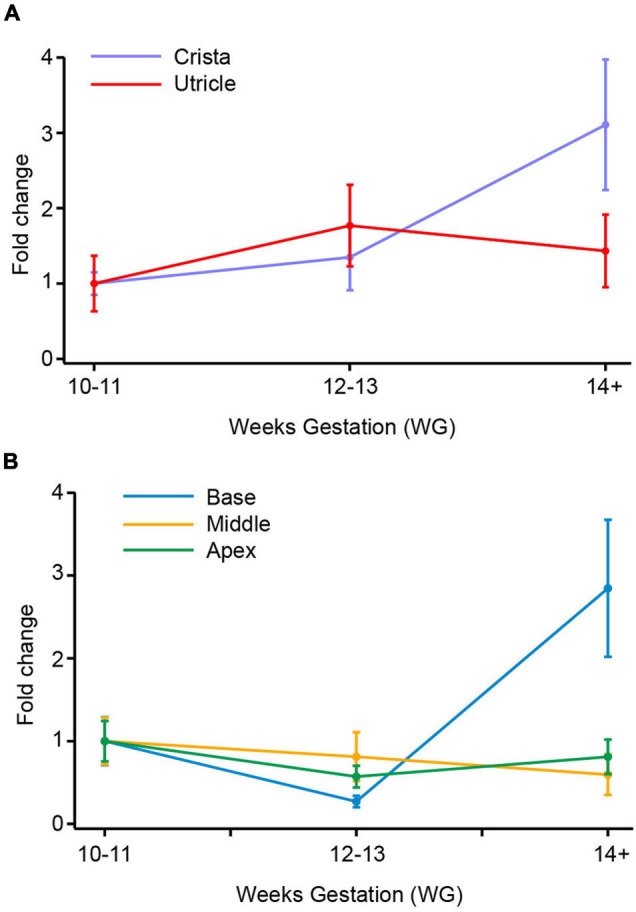
SCN5A (Nav1.5) mRNA in vestibular organs and cochlea. **(A)** In utricle there is no difference in the expression of SCN5A across the three age groups. In crista, there is a threefold increase in SCN5A from 10–11 WG to 14+ WG. **(B)** In cochlea there is a 2.8-fold increase in expression of SCN5A mRNA between 10–11 WG and 14+ WG. There were no changes in expression of SCN5A in middle or apex of cochlea.

**FIGURE 2:**
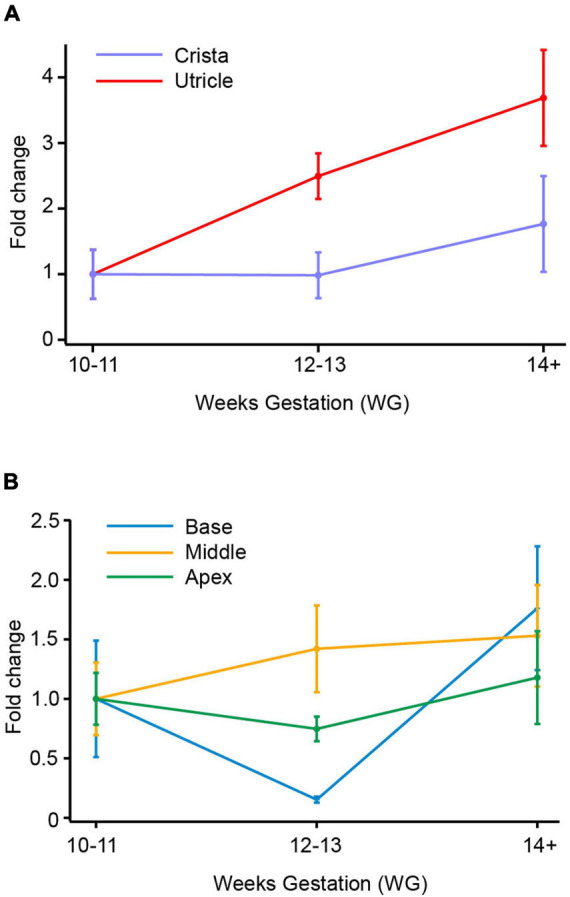
SCN8A (Nav1.6) mRNA in vestibular organs and cochlea. **(A)** Both crista and utricle exhibit an increase in SCN8A mRNA expression with age. At the ages examined there was a peak in fold change at 14+ WG in both crista (1.8-fold) and utricle (3.7-fold). **(B)** There were smaller fold changes in the cochlea compared to vestibular organs. The base and apex of the cochlea show decreases in SCN8A expression at 12–13 WG compared to 10–11 WG. At 14+ WG both base and apex then have increases in mRNA expression, greater than that at 10–11 WG and 12–13 WG. The increase in SCN8A mRNA expression in the base of the cochlea was significantly greater at 14+ WG than at 12–13 WG. In contrast to the base and apex, the middle turn of the cochlea had a slight increase in mRNA expression at 12–13 WG compared to 10–11 WG, which plateaued at 14+ WG.

**FIGURE 3:**
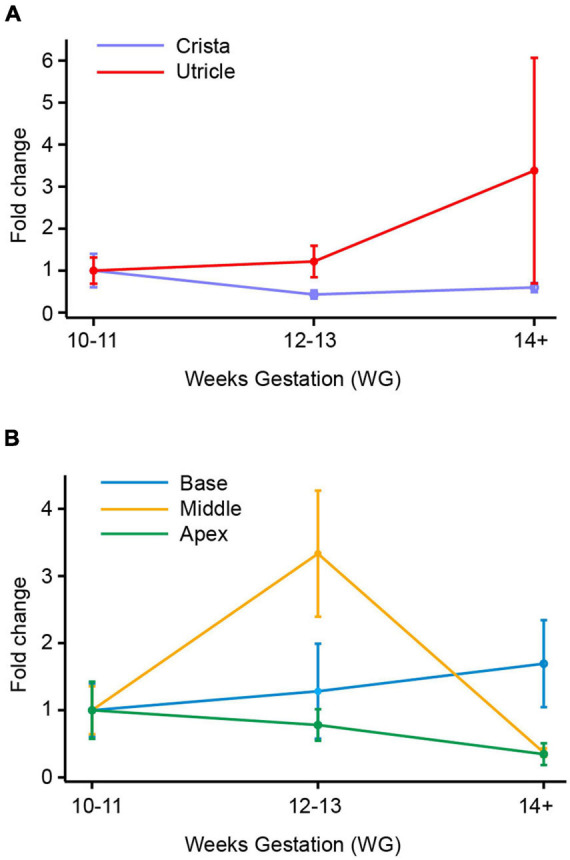
SCN9A (Nav1.7) mRNA in vestibular organs and cochlea. **(A)** There was a slight decrease in SCN9A expression with age in the crista, with the lowest expression level at 12–13 WG (0.4-fold change). In utricle, there was a slight increase in SCN9A expression at 12–13 WG, to peak at 14+ WG with a 3.4-fold change in SCN9A expression relative to the 10–11 WG age group. **(B)** SCN9A expression in the base of the cochlea increased with age to peak at 14+ WG with a 1.7-fold increase compared to 10–11 WG. There was a 3.3-fold increase in SCN9A expression in the middle region of the cochlea at 12–13 WG, which then dramatically decreased at 14+ WG resulting in a 0.4-fold decrease compared to 10–11 WG. There was a decrease in SCN9A expression with age in the apex of the cochlea with lowest expression at 14+ WG (0.006-fold compared to 10–11 WG).

**FIGURE 4:**
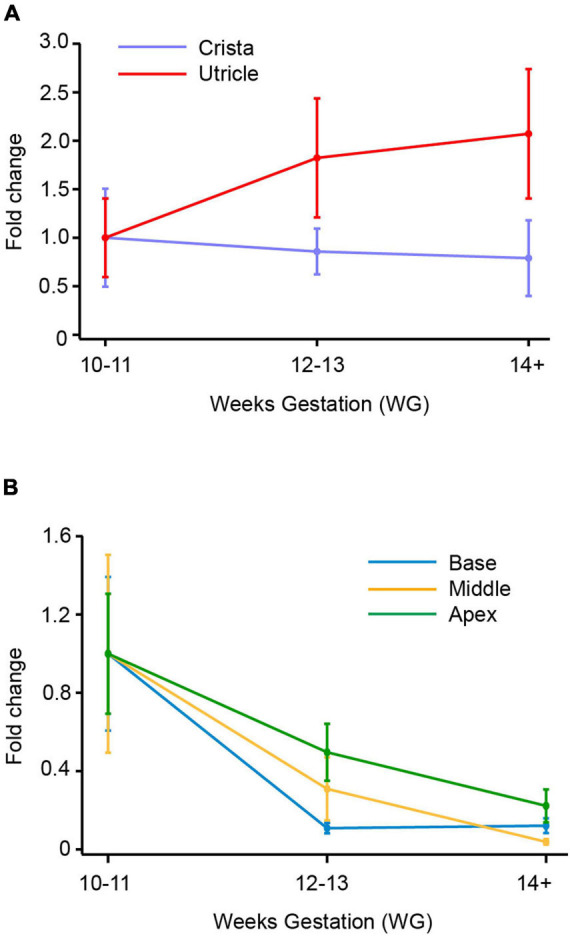
SCN10A (Nav1.8) mRNA in vestibular organs and cochlea. **(A)** In crista there was a decrease in expression of SCN10A between 10–11 WG and 14+ WG. At 14 WG, SCN10A expression was 0.8-fold less than that observed at 10–11 WG. In contrast, in the utricle, SCN10A expression steadily increased to peak at 14+ WG where expression was twofold greater than the youngest age. **(B)** In the base, middle, and apex of the cochlea there was a consistent decrease in expression of SCN10A between 10–11 WG and 14+ WG. SCN10A expression was lowest at 14+ WG within the cochlea, decreasing to 0.003-fold in the middle region of the cochlea.

Gene expression data were collected from inner ear samples aged 10–17 WG from a total of 47 donations. Data were grouped into the following age categories; 10–11, 12–13, and 14+ WG. Changes in mRNA expression of four different Na^+^ channel genes, SCN5A, SCN8A, SCN9A, and SCN10A were tracked during fetal development. These genes were chosen as changes in their expression has been reported in other species during development (Wooltorton et al., 2007; Fryatt et al., 2009; Liu et al., 2016; Zhou et al., 2020). For each gene we first describe ***fold changes*** in gene expression over time for each inner ear region (i.e., crista, utricle, base, middle, and apex of the cochlea, respectively). These fold changes are shown in Figures 1–4 where data points represent the mean fold change ± SEM (i.e., fold change ratio) for the gestational ages shown. We then compare ***relative gene expression*** between vestibular and auditory regions. These data are presented in Tables 2–4.

**TABLE 2:** Relative expression of SCN5A in base of cochlea compared to other inner ear regions at 14+ WG.

**Region**	***N***	**Median**	***U***	**Significance**
Base	8	15.8 × 10^–2^		
Utricle	12	1.5 × 10^–2^	21	0.037*
Crista	9	1.8 × 10^–2^	15	0.043*
Middle	9	0.85 × 10^–2^	14	0.034*
Apex	8	0.45 × 10^–2^	11	0.028*

*p < 0.05.

**TABLE 3:** Relative expression of SCN8A, SCN9A, and SCN10A in utricle compared to cristae ampullares during fetal development.

	**Age**	**Utricle**		**Crista**		***U***	**Significance**	**η^2^**
		**Median**	***N***	**Median**	***N***			
SCN8A	10–11 WG	2.4 × 10^–2^	8	4.0 × 10^–4^	11	0	*p* < 0.001**	0.69
	12–13 WG	3.8 × 10^–2^	11	6.3 × 10^–4^	8	7	*p* = 0.002**	0.49
	14+ WG	8.9 × 10^–2^	11	1.4 × 10^–3^	9	0	*p* < 0.001**	0.70
SCN9A	10–11 WG	10.2 × 10^–3^	7	1.2 × 10^–3^	9	6	*p* = 0.007**	0.45
	12–13 WG	21.0 × 10^–3^	9	1.4 × 10^–3^	8	2	*p* = 0.001**	0.63
	14+ WG	13.3 × 10^–3^	9	1.8 × 10^–3^	8	0	*p* = 0.001*	0.71
SCN10A	10–11 WG	17.4 × 10^–4^	6	1.7 × 10^–4^	4	2	*p* = 0.03*	0.45
	12–13 WG	24.5 × 10^–4^	8	1.3 × 10^–4^	6	4	*p* = 0.01**	0.48
	14+ WG	38.4 × 10^–4^	8	0.4 × 10^–4^	7	0	*p* = 0.001**	0.70

*p < 0.05.

**p < 0.01.

**TABLE 4:** Relative expression of SCN8A in apex compared to base and middle of cochlea at 12–13 WG.

**Apex**		**Middle**		***U***	**Significance**	**η^2^**
**Median**	***N***	**Median**	***N***			
19.3 × 10^–3^	10	6.7 × 10^–3^	8	13	*p* = 0.016*	0.32
		
		**Base**				
		**Median**	***N***	***U***	**Significance**	**η^2^**
		
		9.6 × 10^–3^	8	10	*p* = 0.008**	0.39

*p < 0.05.

**p < 0.01.

### SCN5A (Nav1.5) mRNA Expression

SCN5A gene expression between 10–11 WG and 14+ WG in cristae and utricle were not statistically significantly different (crista; 10–11 WG median = 5.2 × 10^–3^, *n* = 12, 14+ WG median = 1.8 × 10^–2^, *n* = 9, *U* = 31, *p* = 0.102, utricle; 10–11 WG median = 0.02, *n* = 8, 14+ WG median = 0.015, *n* = 12, *U* = 47, *p* = 0.97). There was, however, a threefold change in SCN5A expression between 10–11 WG and 14 WG in crista (Figure 1A). In the base of the cochlea, there was a threefold increase in SCN5A gene expression between 12–13 WG and 14+ WG, and was statistically significantly different. SCN5A expression in the 14+ WG age group (median = 15.8 × 10^–2^, *n* = 8) was significantly greater than the 12–13 WG age group (median = 1.8 × 10^–2^, *n* = 8; *U* = 13, *p* = 0.046) with a large effect size (η^2^ = 0.25). There were no other significant changes in expression of SCN5A with age in the other regions (middle and apex) of the cochlea.

There were several age-dependent differences in the relative expression of SCN5A between inner ear regions. For the vestibular system, the relative expression of SCN5A was significantly higher in the utricle at 10–11 WG (median = 20.7 × 10^–3^, *n* = 8) compared to the cristae (median = 5.2 × 10^–3^, *n* = 12, *U* = 15, *p* = 0.011), but not at other ages. For the cochlea, the relative expression of SCN5A at 14+ WG was significantly greater in the base compared to all other inner ear regions (see Table 2).

### SCN8A (Nav1.6) mRNA Expression

There is a general trend toward an increase in SCN8A gene expression in vestibular organs with age (Figure 2A). This increase in SCN8A expression is significant between 10–11 WG (median = 2.4 × 10^–2^, *n* = 8) and 14+ WG (median = 8.9 × 10^–2^, *n* = 11) in the utricle (*U* = 17, *p* = 0.026) reflecting a large effect size (η^2^ = 0.26). However, there were no differences in the expression of SCN8A in the cristae ampullares with age. But there is a significantly greater relative expression of SCN8A in the utricle compared to the cristae at each age group (see Table 3).

Fold changes in the relative expression of SCN8A in regions of the cochlea are shown in Figure 2B. In the base of the cochlea, there is a decrease in SCN8A expression between 10–11 WG and 12–13 WG, followed by a marked increase in SCN8A expression between 12–13 WG and 14+ WG. This increased SCN8A mRNA expression between 12–13 WG (median = 9.6 × 10^–3^, *n* = 8) and 14+ WG (median 78.7 × 10^–3^, *n* = 8) was statistically significant (*U* = 12, *p* = 0.036), with a large effect size (η^2^ = 0.27). It should be noted that at 14+ WG the increase in SCN8A expression is 1.8-fold greater than that at 10–11 WG, but relative expression between these two ages was not statistically significantly different. There were no changes in SCN8A expression in the middle turn or apex of the cochlea with age. The apex showed a similar aged-related pattern of expression as observed in the base, but the magnitude of the fold changes was not as great and not significant.

At 12–13 WG, the relative expression of SCN8A in the apex was significantly greater than relative expression in both the base and middle regions of the cochlea, in both instances this represents a large effect size (see Table 4). Relative expression of SCN8A was greater in the base, middle, and apex of the cochlea compared to the cristae ampullares at the ages investigated (see Supplementary Tables 1–3). In contrast to the crista, the relative expression of SCN8A was greater in the utricle compared to the middle of the cochlea at 10–11 WG and 14+ WG (see Supplementary Table 4) and the apex of the cochlea at 12–13 WG and 14+ WG (see Supplementary Table 5).

### SCN9A (Nav1.7) mRNA Expression

In the utricle and crista, there are no significant differences in expression of SCN9A expression with gestational age (Figure 3A). There was in the utricle, however, a strong trend toward increased expression of SCN9A between 10–11 WG and 14+ WG (i.e., 3.4-fold increase), but this was not significant. There are significant differences in the relative expression of SCN9A between the two vestibular regions (see Table 3). There were greater levels of expression in the utricle compared to the crista at all three age groups as evidenced by large effect sizes.

SCN9A fold changes in the cochlea are shown in Figure 3B. There were no significant differences in SCN9A expression in the base with age. In the middle region of the cochlea, there is a 3.5-fold increase in SCN9A expression between 10–11 WG and 12–13 WG, but this was not statistically significant. Between 12–13 WG (median = 5.8 × 10^–2^, *n* = 9) and 14+ WG (median = 0.4 × 10^–2^, *n* = 9) there is a significant decrease in SCN9A expression (*U* = 11, *p* = 0.009) with a large effect size (η^2^ = 0.38). In the apex there was a consistent decrease in the expression of SCN9A with age. SCN9A expression at 10–11 WG (median = 17.3 × 10^–3^, *n* = 9) is significantly higher than at 14+ WG (median = 5.0 × 10^–3^, *n* = 8; *U* = 14, *p* = 0.034).

At the two younger age groups, 10–11 WG and 12–13 WG, the cristae ampullares and base of the cochlea had significantly lower relative expression of SCN9A compared to the utricle, middle, and apex of the cochlea.

### SCN10A (Nav1.8) mRNA Expression

There were no significant differences in SCN10A expression with age in either the crista or utricle. The utricle did show a twofold increase in the expression of SCN10A between 10–11 WG and 14+ WG (Figure 4A), but this was not statistically significant. In contrast, the crista showed a 0.8-fold decrease in the expression of SCN10A by 14+ WG (Figure 4A). SCN10A expression was significantly higher in the utricle compared to the crista at each age group (see Table 3).

In the cochlea, SCN10A expression declined between 10–11 WG and 14+ WG in base, middle, and apex (Figure 4B). However, these decreases in SCN10A expression with age in each region of the cochlea were not statistically significant.

The relative expression of SCN10A was significantly greater in the base, middle, and apex of the cochlea compared to the crista during development (see Supplementary Tables 6–8). There were no significant differences in the relative expression of SCN10A between the utricle and regions of the cochlea. Similarly, there were no differences in relative expression of SCN10A between cochlea regions at any age.

### Electrophysiological Recordings

Electrophysiological data were collected from a total of 73 donor samples ranging in age from 10 WG to 16 WG.

### Na^+^ Currents

**FIGURE 5:**
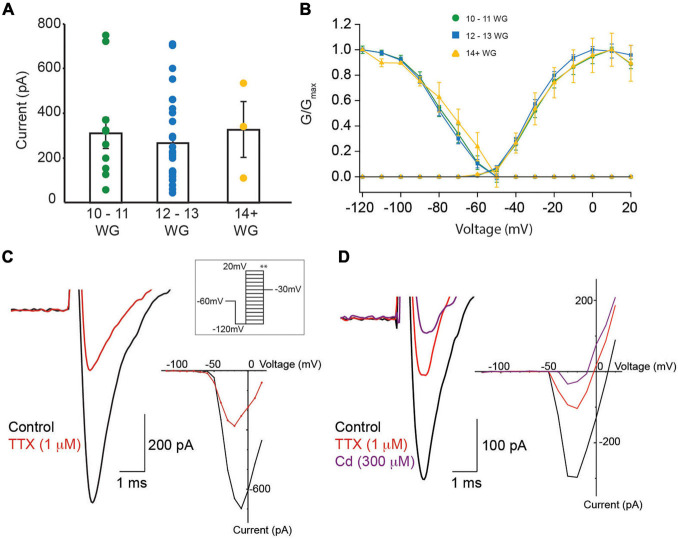
Na^+^ currents in fetal vestibular hair cells. **(A)** The mean Na^+^ current amplitude recorded from vestibular hair cells in three age groups, 10–11 WG, 12–13 WG, and 14+ WG. Scatter of individual Na^+^ current amplitudes for each cell in each age group is shown. **(B)** Activation and inactivation plots for Na^+^ currents for three age groups. **(C)** Left panel: a representative example of a Na^+^ current from hair cell aged 12 WG in control L15 media (black trace) and in the presence of TTX (1 μM; red trace). **(C)** Right panel: the I–V plot for the cell shown in **(C)** *left panel* in control solution (black trace) and in 1 μM TTX (red trace). **(D)** Left panel: a representative example of a Na^+^ current from cristae hair cell aged 11 WG in control L15 media (black trace), in the presence of TTX (1 μM; red trace), and in the presence of both TTX and cadmium (300 μM; purple trace). The Na^+^ current was evoked by stepping from −120 to −20 mV. **(D)** Right panel: the I–V plot for the cell shown in **(D)** *left panel* in control solution (black trace), 1 μM TTX (red trace), and 300 μM cadmium (purple trace). **(A,B)** Mean and SEM are shown.

All recordings were made using a KCl/gluconate based internal solution. From a total of 181 hair cell recordings, ∼30% (*n* = 57) exhibited presumptive Na^+^ currents. Close inspection showed 40 cells were suitable for detailed analysis (17 were removed because of series resistance changes). All cells selected for analysis exhibited whole-cell currents that were consistent with type II vestibular hair cells. The characteristic whole-cell current associated with type I vestibular hair cells, *I*_*k,l*_, was not present in any recorded cells at any age. Sodium current peak amplitude varied markedly (−42.5 to −747.6 pA) across the three age groups. Figure 5A compares mean Na^+^ current amplitude for the three age groups. Mean peak current was similar in the three age groups (10–11 WG = 310.6 ± 69.2 pA, 12–13 WG = 266.8 ± 42.4 pA, and 14+ WG = 326.0 ± 122.7 pA). Na^+^ currents were present in 38% of all hair cells recorded at 10–11 WG and 37% of hair cells aged 12–13 WG. However, in the 14+ WG age group, only three hair cells possessed Na^+^ currents, constituting 11% of all total cells recorded at this age group. Activation and inactivation curves for the three age groups is shown in Figure 5B.

Previous studies have shown two types of Na^+^ current in developing vestibular hair cells, with different inactivation kinetics (*V*_1/2_) (Wooltorton et al., 2007). For inactivation, cells were held at various potentials then stepped to −30 mV (Figure 5C inset ^∗∗^). Na^+^ currents are classified as Na_1_ or Na_2_ when their *V*_1/2_ inactivation, is more negative than −81 mV or more positive than −81 mV, respectively. Based on this classification, the *V*_1/2_ inactivation, *G*_max_, and current slope of Na^+^ currents were obtained during inactivation (Figure 5C inset ^∗∗^). Values for the three age groups are shown in Table 5. We only provide statistics for the 12–13 WG age group as the number of hair cells in other age groups exhibiting Na^+^ currents were too small. For this age group, *V*_1/2_ inactivation for cells classified as Na_1_ (median = 89.7 mV, *n* = 9) was significantly more negative than those classified as Na_2_ (median = 74.5, *n* = 19, *U* = 0, *p* < 0.001). The *G*_max_ and slope values for inactivation were not different. Given the difficulty of obtaining a sufficient number of human fetal hair cells at each age group that possessed Na^+^ currents, we could not further analyze data according to *V*_1/2_ inactivation.

**TABLE 5:** *V*_1/2_ inactivation, *G*_max_, and slope values for Na^+^ currents in response to inactivation protocol.

	***V*_1/2inact_ (mV)**		***G*_max_**		**Slope**	
	**Na_1_**	**Na_2_**	**Na_1_**	**Na_2_**	**Na_1_**	**Na_2_**
10–11 WG	−86.44.0(2)	−73.31.7(9)	4.9 ± 2.0 (2)	4.40.8(9)	6.0 ± 0.5 (2)	7.70.4(9)
12–13 WG	−89.72.5(9)	−74.51.0(19)	4.9 ± 0.8 (9)	3.50.6(19)	6.9 ± 0.7 (9)	7.80.4(19)
14+ WG	−85.00(1)	−72.42.1(2)	1.8 (1)	6.92.1(2)	7.6 (1)	9.50.5(2)

*Number of cells in each age group is shown in brackets ().*

The sensitivity to TTX varies depending on Na^+^ channel type/expression in different tissues (Zimmer, 2010; Marler et al., 2018; de Oliveira et al., 2019) and this variability in TTX sensitivity is also evident in vestibular hair cells (Wooltorton et al., 2007). Accordingly, in a subset of recordings (*n* = 17), we assessed the sensitivity of Na^+^ currents to TTX (1 μM). Our data shows that TTX blocked Na^+^ currents in each age group. Peak Na^+^ current amplitude, half maximal activation (*V*_1/2_), and slope for Na^+^ channel activation, at each age group before and after the application of TTX is shown in Table 6. TTX (1 μM) blocked 60% and 56% of the Na^+^ current, at 10–11 WG (*n* = 6) and 12–13 WG (*n* = 10), respectively. Only one cell was tested in the older group. Figure 5C shows an inward current with a peak current of ∼−700 pA in control solution. This inward current was partially blocked by the Na^+^ channel antagonist TTX (1 μM) in a hair cell aged 12–13 WG (red trace, Figure 5C).

**TABLE 6:** Peak Na^+^ current amplitude, *V*_1/2_, and slope for each age group, before and after, the addition of TTX (1 μM).

**Age**	***n***	**Peak amplitude (pA)**		***V*_1/2_ (mV)**		**Slope**	
		**Control**	**TTX**	**Control**	**TTX**	**Control**	**TTX**
10–11 WG	6	−336.3 ± 85.6	−133.2 ± 37.3	−40.5 ± 2.1	−48.1 ± 3.3	4.8 ± 0.4	4.4 ± 0.8
12–13 WG	10	−341.5 ± 7.3	−191.3 ± 51.7	−38.7 ± 0.8	−50.5 ± 2.8	5.0 ± 0.5	5.0 ± 0.7
14+ WG	1	−531.6	−128.1	−42.9	−75.5	4.3	14.5

Interestingly, in the two younger age groups, 1 μM TTX did not completely block Na^+^ currents, suggesting a component of the inward current in human fetal hair cells is TTX resistant. This somewhat matches a previous report describing TTX resistant Na^+^ currents in vestibular hair cells, albeit using a lower TTX concentration (500 nM versus 1 μM) (Wooltorton et al., 2007). In our study, while 1 μM TTX was found to block a significant proportion of the inward Na^+^ current, there remains a considerable component of the inward current in human fetal hair cells that is TTX resistant. It is possible that in response to depolarization, the residual inward current that remains after TTX exposure is due to Ca^2+^ influx. To test this, we applied the Ca^2+^ channel blocker cadmium (Figure 5D). Cadmium (300 μM) partly blocked the inward current, suggesting some fraction of the inward current in human fetal hair cells may be due to Ca^2+^ influx. However, in some instances, cadmium has also been shown to block a component of Na^+^ currents (Wooltorton et al., 2007). The cadmium-sensitive component accounts for approximately 31% of the total inward current (*n* = 4 cells). Consequently, our results suggest the inward current has three components: TTX-sensitive, TTX-insensitive, and a Ca^2+^ channel component.

## Discussion

Here we targeted four different Na^+^ channel genes, SCN5A, SCN8A, SCN9A, and SCN10A corresponding to Nav1.5, Nav1.6, Nav1.7, and Nav1.8 channels, respectively, which are either TTX sensitive (Nav1.6 and Nav1.7) or TTX insensitive and TTX resistant (Nav1.5 and Nav 1.8, respectively). This is the first time that each of the four genes investigated have been shown to be expressed in human fetal auditory and vestibular neuroepithelium. Due to the difficulty of isolating sufficient tissue for qPCR analysis, across all age fetal groups, Na^+^ channel gene expression was measured from cochlea and vestibular neuroepithelium, that contained a combination of hair cells, afferent, and efferent terminals, and stromal cells. There is a diversity of Na^+^ channel gene expression throughout the body, Nav 1.5 is mainly expressed in cardiac myocytes, while Nav 1.6 expression is primarily in the central nervous system. Nav1.7 and Nav1.8 expression is found in the peripheral nervous system, specifically in dorsal root ganglion neurons and are thought to have a role in nocioception (Tan et al., 2014; Hameed, 2019). The genes investigated in this study were selected based on previous studies which showed expression in both auditory and vestibular systems in other species (Chabbert et al., 1997; Wooltorton et al., 2007; Liu et al., 2016; Zhou et al., 2020).

In all rodents studied to date, there is an initial embryonic and early postnatal developmental expression of Na^+^ channels in hair cells of the vestibular and cochlea neuroepithelium, which declines postnatally (Marcotti et al., 2003; Geleoc et al., 2004; Wooltorton et al., 2007; Meredith and Rennie, 2020). The initial developmental expression was thought to be important for recruiting appropriate afferent connections during development. In the cochlea, IHCs express Na^+^ channels and also spontaneously discharge action potentials, where Na^+^ channel activation is believed to play a role in modulating action potential frequency (Marcotti et al., 2003). In contrast, mouse and rat vestibular hair cells do not spontaneously discharge (Geleoc et al., 2004), and rat utricular hair cells often required a brief hyperpolarization followed by a depolarizing pulse before they fire a single action potential (Wooltorton et al., 2007).

### Tetrodotoxin-Sensitive Na^+^ Channel Expression in Vestibular Neuroepithelium

In rats, genes for six TTX-sensitive Na^+^ channels (Nav1.1, Nav1.2, Nav1.3, Nav 1.4, Nav1.6, and Nav1.7) were reported in utricular epithelium at PND 1 (Mechaly et al., 2005; Wooltorton et al., 2007; Liu et al., 2016). Indeed, there is considerable cell-to-cell variability in the expression of Na^+^ channel subtypes in rat utricular hair cells aged postnatal days 1–2 (Chabbert et al., 2003). However, by PND 21 expression of Nav1.3 and Nav1.4 was absent in rat utricle (Wooltorton et al., 2007). Electrophysiological and anatomical data suggest that Na^+^ channel activity in rat utricular hair cells is confined to the first postnatal week of development (Chabbert et al., 2003), but there is evidence for Na^+^ currents in hair cells of the rat crista until 3 weeks and 35 days in gerbil (Li et al., 2010). Within the first week, single cell RT-PCR from rat utricular hair cells report the expression of Nav1.2, Nav1.3, with Nav1.6 having the most prominent Na^+^ channel expression (Chabbert et al., 2003). Immunolabelling studies show Nav1.6 localized to afferent fibers (Lysakowski et al., 2011) and physiological studies show Nav1.6 is present in mature calyx afferent terminals rather than immature terminals (Meredith and Rennie, 2018). In particular, Nav1.6 is more highly expressed in peripheral zone calyces that are responsible for high firing rates and regular discharge (Meredith and Rennie, 2020). In human fetal epithelium, our qPCR data shows a 3.7 and 1.7-fold-increase in SCN8A (Nav1.6) expression in utricle and crista, respectively, between 10–11 WG and 14+ WG (see Figure 2). Expression of another TTX-sensitive subunit gene, SCN9A (Nav1.7) also showed a threefold increase in expression between 10–11 WG and 14+ WG, but only in the utricle (Figure 3). Functionally, at both the younger ages of development investigated (10–11 WG and 12–13 WG), hair cells with Na^+^ currents have a significant proportion that is blocked by the application of TTX (1 μM). This supports the notion that the TTX sensitive component is due to either Nav1.6 or Nav1.7 channels expressed in human fetal vestibular hair cells.

### Tetrodotoxin-Insensitive/Resistant Na^+^ Channel Expression in Vestibular Neuroepithelium

Embryonic mouse utricular hair cells possess TTX-resistant Na^+^ currents, that peak at embryonic day 16 and are absent at birth (Geleoc et al., 2004). Likewise in rat, TTX-resistant Nav1.8/Nav1.9 channels are absent from utricle at PND 1 (Mechaly et al., 2005; Wooltorton et al., 2007). The gene for the TTX-insensitive channel, Nav1.5 is expressed at PND 1 and PND 21 in utricle (Wooltorton et al., 2007). Nav1.5 has been shown to be localized to the inner face of calyceal terminals, particularly in the striola and may have a role establishing zonal differences in discharge regularity (Lysakowski et al., 2011). Our qPCR data suggest the presence of both TTX-insensitive, Nav1.5 and TTX-resistant, Nav1.8, Na^+^ channel subtypes within the vestibular neuroepithelium during human fetal development. Electrophysiological recordings show the persistence of Na^+^ channel activation in hair cells in the presence of TTX. At 10–11 WG and 12–13 WG, approximately 40% of the inward current remained in the presence of 1 μM TTX, a concentration that blocks TTX-sensitive Na^+^ subunits (typically blocked by 50 nM TTX) and the TTX-insensitive subunit, Nav1.5 (typically blocked by 500 nM TTX). Although we did not attempt to further pharmacologically dissect the contribution of Nav1.5 subunits to the Na^+^ current, our qPCR data suggests an increased contribution of Nav1.5 to the TTX-insensitive component of the Na^+^ current. We showed a 3.1-fold-change increase in expression of SCN5A in crista between 10–11 WG and 14+ WG (see Figure 1). TTX-resistant Na^+^ currents have also been recorded in mammalian vestibular hair cells (Rüsch and Eatock, 1997; Geleoc et al., 2004). In immature gerbil calyceal afferent terminals and vestibular hair cells, the application of 1 μM TTX reveals both TTX-sensitive and TTX-resistant Na^+^ currents, with the TTX-resistant component likely due to expression of Nav 1.8 channels (Meredith and Rennie, 2018). Similarly, in human fetal vestibular hair cells the remaining TTX-resistant component is likely due to expression of Nav1.8. However, our qPCR data shows an increase in SCN10A (Nav1.8) expression in the utricle during fetal development but a concomitant decrease in SCN10A expression in the crista during the same period. One reason for the discrepancy in SCN10A expression with age, between utricle and crista may be that our utricular dissections inadvertently included nearby vestibular ganglion neurons, which have been shown to express Nav1.8 subunits (Liu et al., 2016).

### Na^+^ Channel Characteristics in Vestibular Epithelium

Na^+^ channel activation and inactivation parameters have been measured in rat, mouse, and gerbil vestibular periphery (Geleoc et al., 2004; Wooltorton et al., 2007; Liu et al., 2016; Meredith and Rennie, 2018). To compare *G*_max_, *V*_1/2_ and slope for the Na^+^ current inactivation to previous studies, we classified cells according to *V*_1/2_ inactivation; Na_1_ < 81 mV and Na_2_ ≥ 81 mV (Wooltorton et al., 2007). The *V*_1/2_ inactivation of human fetal hair cells at 12–13 WG classified as Na_2_ (∼−73 mV) was similar to that obtained in immature rat hair cells (∼−74 mV), while human fetal hair cells classified as Na_1_ (∼−86 mV) had slightly more positive *V*_1/2_ inactivation than previously reported in mouse (−88 mV; Geleoc et al., 2004), rat (−92 mV; Wooltorton et al., 2007), and gerbil hair cells (−90 mV; Li et al., 2010), but more negative than another study in rat (−80 mV; Chabbert et al., 2003). In rat, Na_1_ was TTX-insensitive and expressed by all type I hair cells (Wooltorton et al., 2007). Our data suggest that Na^+^ currents in developing human vestibular hair cells are comparable to those in immature rat at early stages of development and hair cells with Na_1_ may even be an early indicator of a type I hair cell. The presence of Na^+^ currents in hair cells, at least from 10 WG, also indicates that these currents have a significant role during development. Na^+^ channel associated action potentials are thought to be responsible for release of brain derived neurotrophic factor (BDNF) in rat utricle at P0 (Chabbert et al., 2003). BDNF is proposed to have a role in establishing appropriate afferent neural connections and synaptogenesis.

Previous studies have shown a down regulation of Na^+^ currents with postnatal age (Chabbert et al., 2003; Geleoc et al., 2004; Wooltorton et al., 2007; Li et al., 2010). In human fetal neuroepithelium, the incidence of hair cells with Na^+^ currents decrease with age: from 38% of all recorded cells at 10 −11 WG to 11% of all recorded hair cells at 14+ WG. This suggests there is a similar decline in Na^+^ channel expression in human hair cells, *in utero*. It has been suggested that cell specific Na^+^ currents may have a function in establishing appropriate afferent connections for either bouton terminal or simple or complex calyces (Wooltorton et al., 2007). This would mean, by 14+ WG, the role of Na^+^ currents in vestibular hair cell development is nearing an end. This notion is supported by our recordings of the first indisputable electrophysiological activity from human calyceal terminals at 14 WG (Lim et al., 2014). By this time in development, calyces had formed and presumably made appropriate connections. For technical reasons, we were unable to record from developing human calyceal terminals prior to 14 WG and therefore could not confirm the presence of Na^+^ channel expression as they developed their connections. Our molecular data, however, suggest there are increases in several Na^+^ channel genes throughout the time period examined, which is consistent with a continuing role in Na^+^ channel expression in afferent terminals and fibers.

### Na^+^ Channel Expression in Cochlea Neuroepithelium

In immature mice (P3–P7) genes for six TTX-sensitive Na^+^ channels were detected with different abundance in inner and OHCs, Nav1.1, Nav1.2, Nav1.3, Nav1.4, Nav1.6, and Nav1.7, while genes for TTX-insensitive Na^+^ channels, Nav1.5, Nav1.8, and Nav1.9 were also detected but with much lower relative expression (Zhou et al., 2020). In human fetal cochlea neuroepithelium, we detected expression of genes for Nav1.5, Nav1.6, Nav1.7, and Nav1.8 from 10 WG through to 14+ WG. Additional experiments are required to determine of other subunits are also conserved in human cochlea and to establish the cell expression of these genes.

### Tetrodotoxin-Sensitive Na^+^ Channel Genes SCN8A and SCN9A in the Cochlea

For the TTX-sensitive channel Na^+^ gene SCN8A (Nav1.6), there is a general trend toward fold-change increases in each region of the cochlea between 10–11 WG and 14+ WG, but particularly between 12–13 WG and 14+ WG at the base of the cochlea. In addition to these fold-change increases relative to the 10–11 WG age group, our data also shows a greater relative expression of SCN8A in the apex of the cochlea compared to the base and middle regions at 12–13 WG. Similarly, microarray analysis of various ion channel genes in mice describes greater expression of SCN8A in the apex of the cochlea than the middle and base regions (Yoshimura et al., 2014). It should be noted that there is greater relative expression of SCN8A in base, middle, and apex of the cochlea compared to the crista, but there is similar relative expression to the utricle.

Expression of the other TTX-sensitive gene, SCN9A (Nav1.7) in human fetal cochlea shows a downward trend between 10–11 WG and 14+ WG in the apex of the cochlea, while at the base of the cochlea, the trend is reversed and there is an increased expression with age. At the two younger age groups, SCN9A relative expression in the base is significantly less than the middle and apex of the cochlea. In the middle region of the cochlea there is a peak in SCN9A expression at 12–13 WG, followed by a decline in relative expression. Taken together, this expression pattern suggests an apex-to-base tonotopic gradient of expression. Relative expression of SCN9A may have already peaked in the apex resulting in a consistent decrease with age, while the middle region peaks at 12–13 WG, followed by a decrease in relative expression. During the same time period, SCN9A relative expression in the base increases with age. This would contrast with the base-to-apex tonotopic differentiation that occurs in human cochlea hair cells (Pujol and Lavigne-Rebillard, 1985; Locher et al., 2013). IHCs at the base of the cochlea first differentiate from 12 WG and differentiation continues along the tonotopic access toward the middle and apex by 14 WG, while three rows of OHCs have differentiated at the base at 14 WG (Locher et al., 2013).

However, a tonotopic gradient of expression for at least one other ion channel (Cav1.3) and calcium binding proteins show a similar apex-to-base expression (reviewed in Mann and Kelley, 2011). To our knowledge there are no studies describing the tonotopic gradient for Na^+^ channel genes in any mammal.

Our data in human fetal cochlea are similar to results in developing mouse cochlea, which found SCN9A to have the highest expression of all Na^+^ channels genes (Zhou et al., 2020). Tonotopy of SCN9A expression was evident between mouse IHCs and OHCs, with greater SCN9A relative expression in IHCs in the apex of the cochlea compared to the base, while in OHCs, the reverse was true, with greater SCN9A relative expression in the base (Zhou et al., 2020). In this study we were not able to make comparisons between the two cochlear hair cell types.

Electrophysiological studies in the cochlea suggest the vast majority of Na^+^ currents in IHCs and OHCs are TTX-sensitive (Marcotti et al., 2003; Zhou et al., 2020). In mice, the most predominant Na^+^ channel gene encoded is Nav1.7, a TTX-sensitive channel (Zhou et al., 2020). Interestingly, the sensitivity to TTX differed significantly between the two hair cell types. Na^+^ currents in IHCs were blocked by 1 μM TTX, while OHCs required 10 μM TTX (Zhou et al., 2020). Another study, also in mice reports the *K*_*d*_ for TTX in IHC Na^+^ currents was 4.8 nM, suggesting Nav1.7 as the predominant Na^+^ channel in IHCs (Marcotti et al., 2003). Our data support the presence of both SCN8A and SCN9A Na^+^ channel genes (Nav1.6 and Nav1.7 channels, respectively) in developing human cochlea neuroepithelium. Future electrophysiological studies will be needed to: determine whether human fetal IHCs and OHCs also have differing TTX sensitivity and to establish which Na^+^ channel gene predominates in each hair cell type.

### Tetrodotoxin-Insensitive Na^+^ Channel Genes SCN5A and SCN10A in the Cochlea

Compared to TTX-sensitive SCN genes, there is a consistent decline in the TTX-insensitive/resistant Na^+^ channel genes SCN5A (Nav1.5) and SCN10A (Nav1.8) in cochlea neuroepithelium during human fetal development, with the exception of a peak 2.8-fold-change increase in relative expression of Nav1.5 in the base of the cochlea at 14+ WG compared to 10–11 WG.

In mouse cochlea epithelia, the expression of SCN5A was greater in the apex compared to the base during early postnatal development (Zhou et al., 2020). In contrast to these mouse data, in fetal cochlea neuroepithelium, there were no differences in SCN5A expression levels between cochlea regions, until 14+ WG. At this stage, relative expression in the base was significantly greater than that recorded in middle or apex of the cochlea. It is possible that these are species differences or alternatively, it could be due to the different developmental plan and timeline. With the exception of Zhou et al. (2020), these TTX-insensitive/resistant Na^+^ channel genes have not been investigated in the cochlea – due in part to their low expression levels (Fryatt et al., 2009). Our results, however, show that these two Na^+^ channel genes are expressed in the cochlea epithelium during human fetal development, albeit briefly. The relative expression of SCN5A at 14 + WG is significantly higher in the cochlea base, than all other inner ear regions including the utricle and crista. Similarly, the relative expression of SCN10A is greater in all cochlea regions compared to the crista at 12–13 WG and 14+ WG. It is possible that the transient expression of SCN5A and SCN10A is necessary for establishing appropriate neural connections in specific cell types or may have a tonotopic gradient profile that was not evident during the timeframe investigated. Alternately, these genes may have a more important role during human development than in other species.

## Conclusion

The expression of Na^+^ channels in hair cells of the developing human inner ear is transient, but coincides with a critical period when afferent connections and synapses are being formed. This study has identified different patterns of expression of four Na^+^ channel genes in vestibular and cochlear neuroepithelium during human fetal development and has characterized Na^+^ currents in human vestibular hair cells. We show significant differences in gene expression with age and between different inner ear regions. In particular, our data in human fetal inner ear shows there is likely a greater role for TTX-sensitive Nav1.6 (SCN8A) Na^+^ channels in vestibular neuroepithelium, while Nav1.7 (SCN9A) has a greater contribution in cochlea neuroepithelium. Our data also suggest there are tonotopy-related differences in the expression of Nav1.7 in the cochlea. In contrast, regional differences in gene expression in vestibular organs such as, *central* versus *peripheral* in the crista and *striola* versus *extrastriola* in the utricle were not investigated due to technical limitations of differentiating between these regions in developing neuroepithelium. This was made more difficult because patterns of “mature” markers of these regions (e.g., calbindin and calretinin) are not yet established.

Another noted difference in Na^+^ channel gene expression between vestibular and cochlear neuroepithelium was the presence of TTX-insensitive Na^+^ channel genes, SCN5A and SCN10A. Results suggest SCN5A and SCN10 contribute to a proportion of the Na^+^ current in developing vestibular hair cells. In contrast, in developing human cochlea there is predominately a down-regulation of SCN5A and SCN10A with age. In other species, there is little evidence for either of these two genes in cochlea development.

There is a transient expression of Na^+^ channels in hair cells of the inner ear of several mammalian species (Chabbert et al., 2003; Marcotti et al., 2003; Geleoc et al., 2004; Wooltorton et al., 2007). Our results suggest Na^+^ channel expression in human fetal vestibular and cochlea neuroepithelia are consistent with those from other species. The brief expression of Na^+^ channels in hair cells may have a similar purported role in establishing appropriate afferent contacts and synapse formation. Therefore, these two processes are critical during development to establish normal function. The onset and continued expression of Na^+^ channels in afferent terminals and fibers are essential for the transmission of auditory and vestibular information. During human fetal development, vestibular and auditory reflexes which are indicative of functional afferent activity have been recorded at 19 WG and 24 WG, respectively (Humphrey, 1964; Lecanuet and Schaal, 1996).

Understanding the significant differences and commonalities in expression and timing between the vestibular and auditory systems in human tissue is essential if we are to incorporate appropriate and representative hair cells in human stem-cell derived inner ear organoids (Koehler et al., 2017; Jeong et al., 2018; Lahlou et al., 2018; Mattei et al., 2019). Our current understanding of the chronology of hair cell development and maturation arises primarily from studies in animals. Animal models, such as mice, have helped us understand basic principles, however, their compressed developmental timeline means the often, brief expression of some transcription factors means their functional significance is not always clear. Using human fetal vestibular and cochlear tissue to determine which genes are expressed, and when, and for how long, in each inner ear organ is needed to establish the precise timeline of gene expression and fundamental information for driving potential regenerative technologies that are optimized specifically for humans.

## Data Availability Statement

The raw data supporting the conclusions of this article will be made available by the authors, without undue reservation.

## Ethics Statement

The studies involving tissue donation made by human participants were reviewed and approved by the University of Newcastle Human Research Ethics Committee. The patients/participants provided their written informed consent to participate in this study.

## Author Contributions

RQ, HD, EC, and RL completed the data collection and analysis. MT and BN provided manuscript feedback. RC, AB, and RL conceptualized the project and wrote the manuscript. All authors contributed to the article and approved the submitted version.

## Conflict of Interest

The authors declare that the research was conducted in the absence of any commercial or financial relationships that could be construed as a potential conflict of interest.

## Publisher’s Note

All claims expressed in this article are solely those of the authors and do not necessarily represent those of their affiliated organizations, or those of the publisher, the editors and the reviewers. Any product that may be evaluated in this article, or claim that may be made by its manufacturer, is not guaranteed or endorsed by the publisher.
