# Supplementary material for: Expression and Physiology of Voltage-Gated Sodium Channels in Developing Human Inner Ear
Source: Front Neurosci. 2021 Oct 25;15:733291. doi: 10.3389/fnins.2021.733291 (PMC8575412; doi:10.3389/fnins.2021.733291)
Supplement: Supplementary file 1 [file Table_1.docx]

**Supplementary Table 1. Relative expression of SCN8A in crista compared to cochlea base during fetal development**

| **Age** | **Crista** | | **Base** | | **U** | **Significance** | **η^2^** |
| --- | --- | --- | --- | --- | --- | --- | --- |
|  | **Median** | **N** | **Median** | **N** |  |  |  |
| 10 – 11 WG | 0.4 x 10^-3^ | 11 | 21.7 x 10^-3^ | 11 | 9 | p=0.001** | 0.52 |
| 12 – 13 WG | 0.6 x 10^-3^ | 8 | 9.6 x 10^-3^ | 8 | 2 | p = 0.002** | 0.62 |
| 14+ WG | 1.4 x 10^-3^ | 9 | 78.7 x 10^-3^ | 8 | 2 | p=0.001** | 0.63 |

**** p<0.01**

**Supplementary Table 2. Relative expression of SCN8A in crista compared to cochlea middle during fetal development**

| **Age** | **Crista** | | **Middle** | | **U** | **Significance** | **η^2^** |
| --- | --- | --- | --- | --- | --- | --- | --- |
|  | **Median** | **N** | **Median** | **N** |  |  |  |
| 10 – 11 WG | 0.4 x 10^-3^ | 11 | 5.4 x 10^-3^ | 11 | 11 | p=0.001** | 0.48 |
| 12 – 13 WG | 0.6 x 10^-3^ | 8 | 6.7 x 10^-3^ | 8 | 2 | p = 0.002** | 0.62 |
| 14+ WG | 14.0 x 10^-4^ | 9 | 9.1 x 10^-3^ | 9 | 5 | p=0.002** | 0.54 |

**** p<0.01**

**Supplementary Table 3. Relative expression of SCN8A in crista compared to cochlea apex during fetal development**

| **Age** | **Crista** | | **Apex** | | **U** | **Significance** | **η^2^** |
| --- | --- | --- | --- | --- | --- | --- | --- |
|  | **Median** | **N** | **Median** | **N** |  |  |  |
| 10 – 11 WG | 0.4 x 10^-3^ | 11 | 18.4 x 10^-3^ | 12 | 2 | p<0.001** | 0.67 |
| 12 – 13 WG | 0.6 x 10^-3^ | 8 | 19.6 x 10^-3^ | 10 | 0 | p < 0.001** | 0.70 |
| 14+ WG | 1.4 x 10^-3^ | 9 | 19.8 x 10^-3^ | 8 | 2 | p=0.001** | 0.62 |

**** p<0.01**

**Supplementary Table 4. Relative expression of SCN8A in utricle compared to cochlea middle during fetal development**

| **Age** | **Utricle** | | **Middle** | | **U** | **Significance** | **η^2^** |
| --- | --- | --- | --- | --- | --- | --- | --- |
|  | **Median** | **N** | **Median** | **N** |  |  |  |
| 10 – 11 WG | 23.6 x 10^-3^ | 8 | 5.4 x 10^-3^ | 11 | 12 | p=0.005** | 0.38 |
| 14+ WG | 88.7 x 10^-3^ | 11 | 9.1 x 10^-3^ | 9 | 2 | p<0.001** | 0.65 |

**** p<0.01**

**Supplementary Table 5. Relative expression of SCN8A in utricle compared to cochlea apex during fetal development**

| **Age** | **Utricle** | | **Apex** | | **U** | **Significance** | **η^2^** |
| --- | --- | --- | --- | --- | --- | --- | --- |
|  | **Median** | **N** | **Median** | **N** |  |  |  |
| 12 – 13 WG | 37.9 x 10^-3^ | 11 | 19.6 x 10^-3^ | 10 | 10 | p = 0.002** | 0.48 |
| 14+ WG | 88.7 x 10^-3^ | 11 | 19.8 x 10^-3^ | 8 | 15 | p=0.017* | 0.30 |

*** p<0.05, ** p<0.01**

**Supplementary Table 6. Relative expression of SCN10A in crista compared to cochlea base during fetal development**

| **Age** | **Crista** | | **Base** | | **U** | **Significance** | **η^2^** |
| --- | --- | --- | --- | --- | --- | --- | --- |
|  | **Median** | **N** | **Median** | **N** |  |  |  |
| 10 – 11 WG | 1.7 x 10^-4^ | 4 | 163.0 x 10^-4^ | 12 | 6 | p = 0.03* | 0.30 |
| 12 – 13 WG | 1.3 x 10^-4^ | 6 | 47.5 x 10^-4^ | 9 | 2 | p = 0.003** | 0.58 |
| 14+ WG | 0.4 x 10^-4^ | 7 | 42.8 x 10^-4^ | 8 | 2 | p = 0.003** | 0.60 |

*** p<0.05, ** p<0.01**

**Supplementary Table 7. Relative expression of SCN10A in crista compared to cochlea middle during fetal development**

| **Age** | **Crista** | | **Middle** | | **U** | **Significance** | **η^2^** |
| --- | --- | --- | --- | --- | --- | --- | --- |
|  | **Median** | **N** | **Median** | **N** |  |  |  |
| 12 – 13 WG | 1.3 x 10^-4^ | 6 | 48.7 x 10^-4^ | 8 | 2 | p = 0.005** | 0.58 |
| 14+ WG | 0.4 x 10^-4^ | 7 | 7.1 x 10^-4^ | 9 | 6 | p = 0.007** | 0.46 |

*** p<0.05, ** p<0.01**

**Supplementary Table 8. Relative expression of SCN10A in crista compared to cochlea apex during fetal development**

| **Age** | **Crista** | | **Apex** | | **U** | **Significance** | **η^2^** |
| --- | --- | --- | --- | --- | --- | --- | --- |
|  | **Median** | **N** | **Median** | **N** |  |  |  |
| 12 – 13 WG | 1.3 x 10^-4^ | 6 | 47.0 x 10^-4^ | 8 | 0 | p = 0.002** | 0.69 |
| 14+ WG | 0.4 x 10^-4^ | 7 | 28.0 x 10^-4^ | 8 | 3 | p = 0.004** | 0.56 |

*** p<0.05, ** p<0.01**
